# Development of Micropatterns on Curved Surfaces Using Two-Step Ultrasonic Forming

**DOI:** 10.3390/mi10100654

**Published:** 2019-09-28

**Authors:** Jong-Han Park, Keun Park

**Affiliations:** 1 Department of Mechanical System Design Engineering, Seoul National University of Science and Technology, Seoul 01811, Korea; highpjh@rastech.co.kr; 2 R&D Department, Rastech Co. Ltd., Daejeon 34037, Korea

**Keywords:** micropattern, ultrasonic imprinting, ultrasonic stretching, functional surface, curved surface

## Abstract

Nanoimprint lithography (NIL) is a micro/nanoscale patterning technology on thermoplastic polymer films, and has been widely used to fabricate functional micro/nanoscale patterns. NIL was also used to develop micro/nanoscale patterns on curved surfaces by employing flexible polymer stamps or micropatterned metal molds with macroscopic curvatures. In this study, two-step ultrasonic forming was used to develop micropatterns on a curved surface out of a flat metal stamp, by connecting ultrasonic imprinting and stretching processes. Ultrasonic imprinting was used to replicate functional micropatterns on a flat polymer film, using a flat ultrasonic horn and micropatterned metal stamps with prism and dot micropatterns. An ultrasonic stretching process was then used to form a curvature on the patterned film using a curved ultrasonic horn and a soft mold insert, to avoid damage to the pre-developed micropatterns. The ultrasonic horn was designed to have three different tip radii, and the resulting forming depth and curvature formation were investigated experimentally. As a result, three different curved surfaces containing two different micropatterns were obtained. The developed curved films containing micropatterns were then evaluated optically, and showed different optical diffusion and illumination characteristics according to the film curvature and micropattern type. These results indicate that the proposed technology can extend the functionality of conventional micropatterned products by imposing appropriate curvatures.

## 1. Introduction

In recent decades, functional biological surfaces have received increasing attention [1]. Multiple studies have been performed to understand the nature of biological surfaces and to mimic their special functions, including the superhydrophobic function of lotus leaves [2], the antireflection function of moth eyes [3], the antifogging function of mosquito eyes [4], and the water-harvesting function of the Namib Desert beetles [5]. To fabricate these biomimetic surfaces artificially, micropatterning technologies have been widely used to replicate microscale or nanoscale patterns on the surface of films or substrates.

Nanoimprint lithography (NIL) is a micro/nanoscale technology for patterning polymer films or substrates, which ensures productivity sufficient for mass production [6]. NIL can generally be categorized into two processes, namely, hot embossing lithography (HEL) and UV-based nanoimprint lithography (UV-NIL). HEL uses preheated molds to replicate micro/nanoscale patterns on thermoplastic polymer substrates [7]. Although this process has advantages of simple equipment setup and low cost, its cycle time is too long to be used for mass production [8]. In contrast, UV-NIL uses UV-curable resins and UV-transparent molds to transfer micro/nanoscale patterns [9], and was be applied to mass production by employing a roll-to-roll type forming system [10]. Recently, ultrasonic imprinting was developed to replicate micro/nanoscale patterns on flat polymer surfaces using ultrasonic vibration energy [11,12,13,14,15,16]. Ultrasonic imprinting has the advantage of localized heating capability, and has been employed for selective micropattern replication [17,18,19] and for the development of composite micropatterns [20].

While these micro/nanoscale patterning technologies have been generally used to replicate micropatterns on a flat polymer surface, micropatterning on a curved surface was also developed to enhance functionality of the patterned surface [21]. Various technologies have been used to develop micropatterns on curved surfaces, including microcontact printing [22], step-and-flash imprint lithography [23], soft lithography [24,25,26], vapor-assisted imprinting [27], 3D nanomolding with a thin soft stamp [28], microwrinkling [29], inkjet printing [30], and wrapping of a flexible patterned substrate over a curved object [31]. Among these technologies, the soft lithography or imprinting has been used most popularly owing to its simple and effective process capability, in which flexible polymer stamps are used to replicate micro/nanoscale patterns on curved surfaces [32,33,34].

The use of a flexible polymer stamp, however, has a drawback due to its poor durability under high forming pressure, and hence it has a limitation to being used in mass production [27]. As an alternative solution for mass production, micropatterned metal molds on curved surfaces were fabricated and used in polymer micromolding [35,36]. Although this approach improved the durability of micropatterned molds compared to the flexible polymer stamps, the fabrication of micropatterns on a curved metal surface requires specialized tooling equipment such as a five-axis computerized numeric control (CNC) micromachine.

In this study, an efficient micropatterning technology on a curved surface was developed, without the use of a flexible polymer stamp nor a micropatterned metal mold with a macroscopic curvature. Instead, we added an ultrasonic stretching process to the conventional ultrasonic imprinting process that uses a flat metal stamp only. The ultrasonic imprinting was performed to develop functional micropatterns on a flat polymer film out of the flat metal stamp. The ultrasonic stretching process was then used to form a macroscopic curvature on the patterned film. In this process, an ultrasonic horn with a curved tip and a soft mold insert was used to form a curved surface without damaging the pre-developed micropatterns. Three types of ultrasonic horns with different tip radii were designed and their effects on the curvature formation were investigated experimentally. The optical properties of the developed curved films containing micropatterns were then evaluated, and the effect of the micropattern shapes and curvature were investigated in terms of the light diffusion and illumination characteristics.

## 2. Materials and Methods 

### 2.1. Materials

For the ultrasonic forming, polyethylene terephthalate (PET) films (Skyrol SG00, SK Chemicals Co. Ltd., Seongnam, Korea) were used. The thickness and density of the PET film were 0.3 mm and 1380 kg/m^3^, respectively. High-strength aluminum alloy (AA7075-T6, Dongyang Aluminum Co. Ltd., Siheung, Korea) was used as a horn material due to its high ultrasonic transmission characteristics. The density, elastic modulus, and tensile strength were 2810 kg/m^3^, 71.7 GPa, and 572 MPa, respectively. As a mold insert for ultrasonic micropatterning, nickel stamps were fabricated for prism and dot patterns as described in Section 2.3., and were assembled with AISI-1045 steel mold bases. Silicone rubber (KE951U, Shin-Etsu Silicone, Tokyo, Japan) was used as a mold insert for ultrasonic forming; its density and elastic modulus were 1140 kg/m^3^ and 0.5 MPa, respectively.

### 2.2. Two-Step Ultrasonic Forming

**Figure 1:**
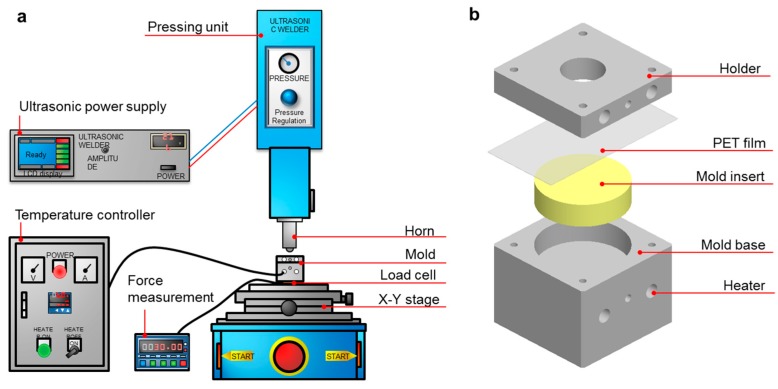
Experimental setup: (**a**) configuration of ultrasonic forming system; (**b**) mold structure for the ultrasonic forming.

The two-step ultrasonic forming process employs ultrasonic imprinting and stretching processes sequentially. The ultrasonic imprinting process is used to develop micropatterns on a flat polymer film. The ultrasonic stretching process is used to form a curvature on the patterned film. Figure 1a show an illustration of the ultrasonic forming system consisting of an ultrasonic generator, a pressing unit and a forming section. An ultrasonic power supply (FST-270A, Forward Sonic Technology Co. Ltd., Siheung, Korea) was used to generate ultrasonic excitation with 700 W power and 27 kHz excitation frequency. A hydraulic pressing unit that supports 1.0 MPa pressure was installed to provide compressive force during the forming process. An ultrasonic horn was attached at the end of the hydraulic unit. Figure 1b illustrates the mold structure where a polymer film is placed between a holder and a mold assembly. The mold assembly consists of a steel mold base and an insert depending on the ultrasonic forming processes, namely, a micropatterned nickel stamp for the ultrasonic imprinting (hard insert) and a silicon rubber insert for the ultrasonic stretching (soft insert).

**Figure 2:**
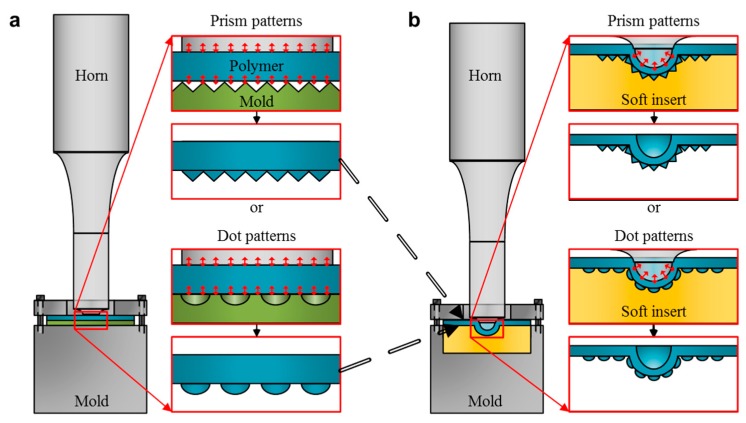
Description of the two-step ultrasonic forming process: (**a**) ultrasonic imprinting; (**b**) ultrasonic stretching.

Figure 2 demonstrates the two-step ultrasonic forming process. At first, ultrasonic imprinting is performed to develop micropatterns, as shown in Figure 2a. A flat horn is placed on a polymer film, and a micropatterned mold is placed under the polymer film (pressing stage). Ultrasonic vibration is transferred to the polymer film through the horn, and the polymer film is heated and softened by repetitive friction with the micropatterned mold (vibration stage). Micropatterns are then replicated on the softened film by the applied pressure during several seconds of holding time (holding stage).

The patterned film is then installed in the stretching mold, and the ultrasonic stretching process is followed. Here, the patterned side is placed on the soft mold insert (silicon rubber) as illustrated in Figure 2b, in order to avoid damage during the ultrasonic stretching process. A curved horn is placed on the patterned film, and pressure is imposed to form an initial curvature in the film (pressing stage). Ultrasonic vibration is then transferred to the patterned film (vibration stage), in order to heat the upper side of the patterned film. After that, holding pressure is imposed to form a permanent curvature in the film without damage in the micropatterns (holding stage).

### 2.3. Fabrication of Micromolds for Ultrasonic Imprinting

**Figure 3:**
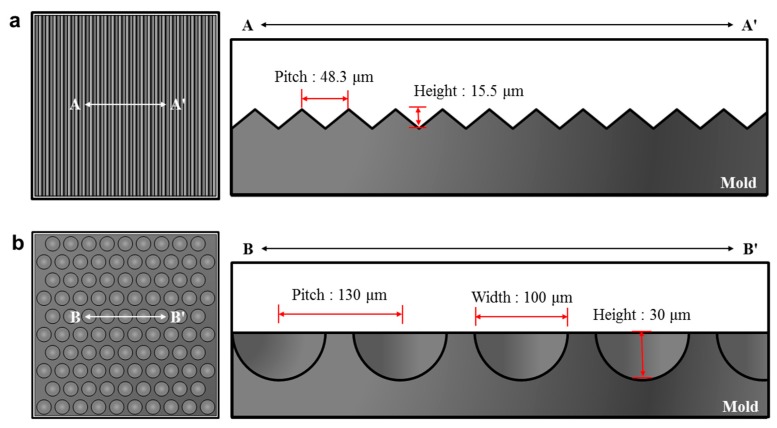
Dimensional configurations of micropatterned mold inserts: (**a**) prism pattern; (**b**) dot pattern.

Two types of micropatterns, prism and dot patterns, were used in ultrasonic imprinting as described in the previous section. Figure 3a shows the dimensional configuration of the prism-patterned mold, of which pitch and height were 48.3 and 15.5 μm, respectively. Figure 3b shows the dimensional configuration of the dot-patterned mold, of which pitch, width, and height were 130, 100, and 30 μm, respectively. 

These patterned mold inserts were fabricated using the MEMS process: photolithography, deep reactive ion etching (DRIE), sputtering, and nickel electroforming processes. After removing the silicon master, a self-assembled monolayer (SAM) was deposited on the surface of the nickel stamp in order to facilitate demolding after imprinting. This mold insert was then assembled with a steel mold base, from which a micromold for ultrasonic imprinting was prepared.

### 2.4. Design and Fabrication of the Ultrasonic Horns

**Figure 4:**
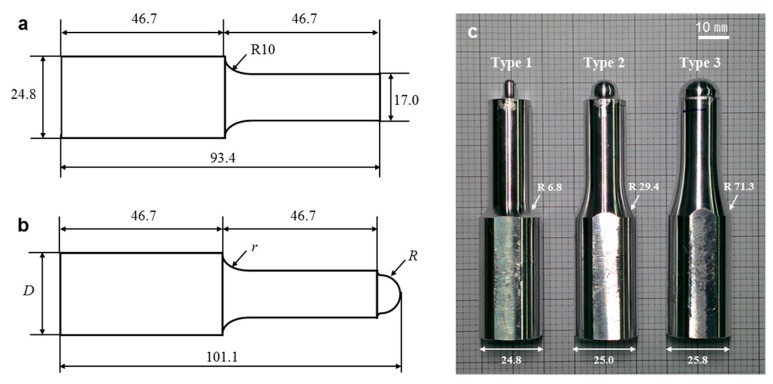
Configuration of the ultrasonic horns: (**a**) flat horn for ultrasonic imprinting; (**b**) curved horn for ultrasonic stretching; (**c**) three design candidates of the curved horn.

For the proposed two-step ultrasonic forming, two ultrasonic horns were required depending on the characteristics of each process, as illustrated in Figure 2. For the ultrasonic imprinting process, a flat-end horn with a circular cross-section was used to press the polymer film and to transfer ultrasonic vibration energy uniformly. The flat horn was designed as shown in Figure 4a, based on a previous study by the authors [37]. 

For the ultrasonic stretching process, on the other hand, the horn was modified to have a curved end which is defined by a single curvature, as shown in Figure 4b. In the curved horn, the horn length and the outlet diameter were set to 101.1 and 17.0 mm, respectively. The radius of the curved end (*R*) was set as a design parameter, and was designed to have three radii (2.5, 5.5, and 8.5 mm) in order to control the curvature of the formed film during the ultrasonic stretching process. The inlet diameter (*D*) and corner radius (*r*) were also set as design parameters to obtain desirable vibration characteristics for different horn tip radii (*R*).

To determine these design parameters, the vibration characteristics of the horns were investigated by finite element (FE) analyses. ANSYS Workbench (ANSYS Inc., Canonsburg, PA, USA) was used to perform the FE analyses. The horns were designed to resonate within a given excitation frequency range (27 ± 0.2 kHz) with a longitudinal vibration mode. The design objectives were then set to obtain their natural frequencies near 27.0 kHz and to maintain the deformation magnitude of the outlet as twice of the inlet deformation (i.e., magnification ratio of 2). The magnification ratio of the horn (*γ*) was defined by the following equation:

(1) $$\gamma =\frac{\delta_{o}}{\delta_{i}},$$

where *δ_i_* and *δ_o_* represent the maximum displacement of the horn at its inlet and outlet, respectively. 

A design of experiment (DOE) was performed to investigate the effects of the design parameters for the three types of horns (*R* = 2.5, 5.5, and 8.5 mm). The DOE was scheduled using the central composite design (CCD) table. For each horn type, nine FE analyses were performed based on the orthogonal array, and the resulting natural frequency (*f*) and magnification ratio (*γ*) were obtained, as provided in the Appendix A. Response surface analysis was then performed and the optimal design parameters were found using the sequential quadratic programming (SQP) method. The resulting optimal dimensions were determined to obtain the desired natural frequency (27,000 Hz) and magnification ratio (2.0). The determined optimal dimensions are listed in Table 1, and the fabricated horns are shown in Figure 4c.

### 2.5. Characterization

The vibration characteristics of the ultrasonic horns were measured by analyzing dynamic displacements of the horn inlet and outlet. A fiber-optic displacement sensor (D63-H1T4Z, PHILTEC Inc., Annapolis, MD, USA) was used to measure dynamic displacements, and the measured signals were recorded using a digital oscilloscope (GDS-1102-IJ, GW Instek Co. Ltd., Taipei, Taiwan). The resonant frequency (*f*) and the horn displacement (*δ*) were determined by analyzing the output signal. Microscopic images were obtained using an optical microscope (Mi-9000, Jason Electro-Tech, Seoul, Korea). The temperature distribution of the polymer film was measured using an infrared thermal imaging system (FLIR E50, FLIR Systems Inc., Boston, MA, USA). A dial gauge (ID-C150B, Mitutoyo Co., Kawasaki, Japan) was used to measure the heights of curved surfaces after ultrasonic stretching. Micropattern heights were measured using a surface roughness tester (Rugosurf 90G, TESA Technology, Renens, Switzerland). 

Optical diffusion and illumination properties were observed to evaluate change in the functionality of a micropatterned film relative to the amount of curvature. The optical diffusion characteristics were analyzed by observing laser light images behind the patterned film. The wavelength, power, and beam diameter of the laser diode were 635 nm, 1 mW, and 3 mm, respectively. The illumination property was analyzed using an imaging colorimeter (ProMetric G3, Radiant Vision Systems LLC, Redmond, WA, USA) by measuring the luminance distribution of an LED light through the micropatterned films.

## 3. Results

### 3.1. Vibration Characteristics of the Ultrasonic Horns

Three fabricated horns were attached to the ultrasonic forming system, and their vibration characteristics were measured using fiber-optic displacement sensors. The measurement results are listed in Table 2 which shows that all fabricated horns successfully resonated in the desired frequency range (27 ± 0.2 kHz). The magnitude of the inlet displacement was measured to 16.4 μm. The outlet displacements of the three horns (*δ_o_*) were measured five times, and the resulting outlet displacements and the corresponding magnification ratios (*γ*) are compared in Table 2. It can be seen that the calculated magnification ratios were near the target value (2.0). These results indicate that the three ultrasonic horns satisfied the design objectives, the resonation frequency range within 27.0 ± 0.2 kHz and magnification of 2.0.

### 3.2. Formability of Curved Surface

**Figure 5:**
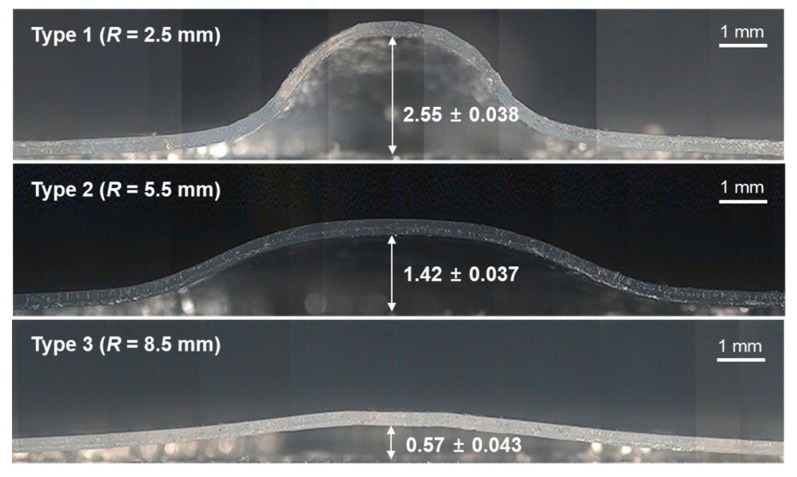
Sectional photographs of the formed film after ultrasonic stretching using various horn types with different tip radii (unit: mm).

To investigate the forming characteristics of the three horns, ultrasonic stretching experiments were performed on plain PET films. The forming pressure and pressing time were set to 0.5 MPa and 4 s, respectively. The ultrasonic vibration time was set to 0.3 s, and the holding time was set to 3 s. Figure 5 shows sectional photographs of the formed surfaces depending on the horn type. The forming heights were measured for ten samples, and the resulting average heights and deviations are marked in Figure 5. It was observed that the forming height increased as the horn radius decreased, showing the largest value (2.55 ± 0.038 mm) when the horn radius was 2.5 mm. In contrast, the forming height was only 0.57 ± 0.043 mm when the horn radius was 8.5 mm.

**Figure 6:**
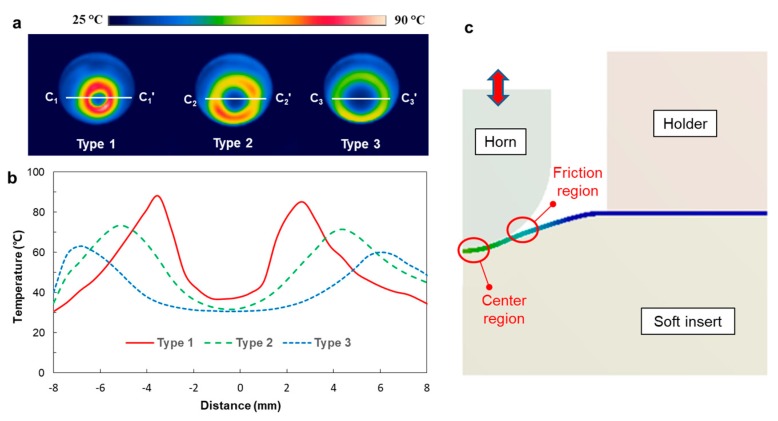
Temperature measurement results after 0.3 s vibration: (**a**) temperature distributions for three horn types; (**b**) comparison of the sectional temperature profiles (Section C_i_–C_i_’ in Figure 6a); (**c**) description of the center region and the friction region.

To analyze this difference in forming height, ultrasonic heating capability was compared for three cases. Figure 6a shows the temperature distributions of the film surfaces after the vibration stage. It can be seen that the high temperature regions have ring shapes in all cases. Type 1 showed the highest temperature. Temperature profiles along the section C_i_–C_i_’ in Figure 6a were compared for three cases, as shown in Figure 6b. In all cases, the temperature profiles showed “M” shapes, showing two peaks near the horn radius and temperature reduction at the center region. This can be explained by the absorption of ultrasonic vibration energy by the soft insert. That is, the center region of the film contacted the soft insert strongly and hence the ultrasonic vibration of the horn did not generate friction between the horn and polymer film. On the other hand, friction occurred at locations where the contact status between the horn and film changed with the vibration of the horn, as illustrated in Figure 6c.

### 3.3. Micropattern Replication Characteristics on Flat Surface

**Figure 7:**
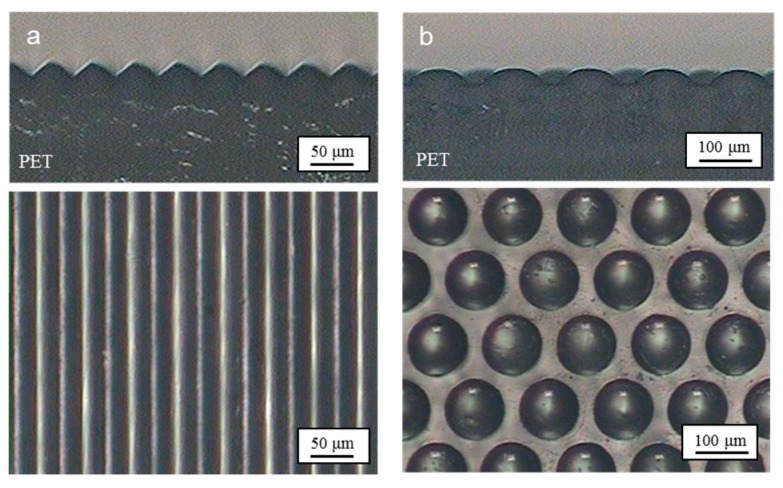
Microscopic images of the replicated micropatterns: (**a**) prism pattern; (**b**) dot pattern.

Ultrasonic imprinting was performed to replicate micropatterns on a flat PET film. Based on the authors’ previous studies [38,39], the ultrasonic vibration time, holding time, and pressure were set to 1 s, 9 s, and 0.4 MPa, respectively. Figure 7a shows microscopic images of the replicated prism patterns. The pattern heights were measured to be 14.8 ± 0.2 μm, which corresponds to 95.5% replication ratio compared to the depth of the micropatterned mold (15.6 μm). Figure 7b shows microscopic images of the replicated dot patterns, the heights of which were measured to be 27.5 ± 0.4 μm. The replication ratio was then calculated to be 93.8% compared to the depth of the micropatterned mold (29.3 μm). These high replication ratios indicate that the micropatterns were replicated successfully under the given imprinting conditions. The patterned films were then used in the following ultrasonic stretching process.

### 3.4. Development of Micropatterns on a Curved Surface

**Figure 8:**
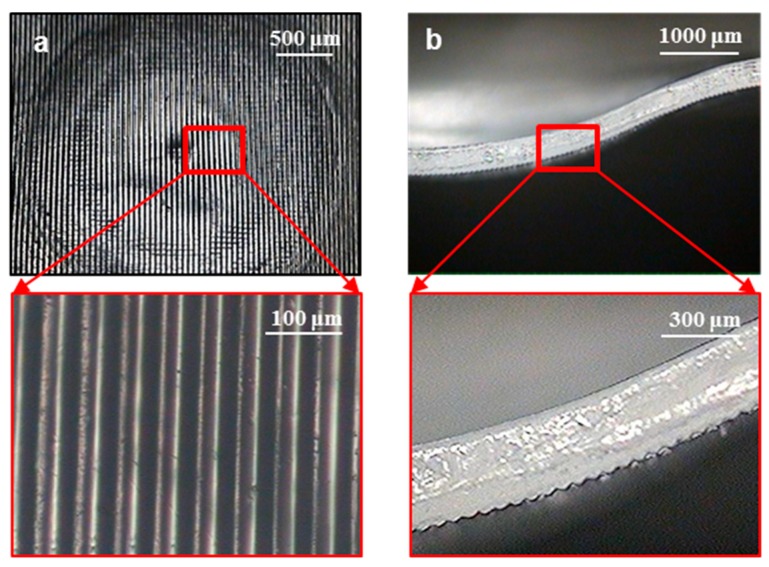
Microscopic images of the prism micropatterns on a curved surface: (**a**) bottom view; (**b**) sectional view.

To develop micropatterns on a curved surface, ultrasonic stretching was performed for the micropatterned PET films. Figure 8 shows microscopic images of the film formed with prism micropatterns, where a Type 2 horn was used for the ultrasonic stretching. It was observed that the micropatterns retained their original shapes after the film was formed into a curved shape. By analyzing the sectional image (Figure 8b), the pattern heights after stretching were measured to be 12.5 ± 0.6 μm. This value corresponds to 84.5% of the pattern height before stretching (14.8 μm), which indicates that the pattern height was reduced by 15.5% during the ultrasonic stretching process.

**Figure 9:**
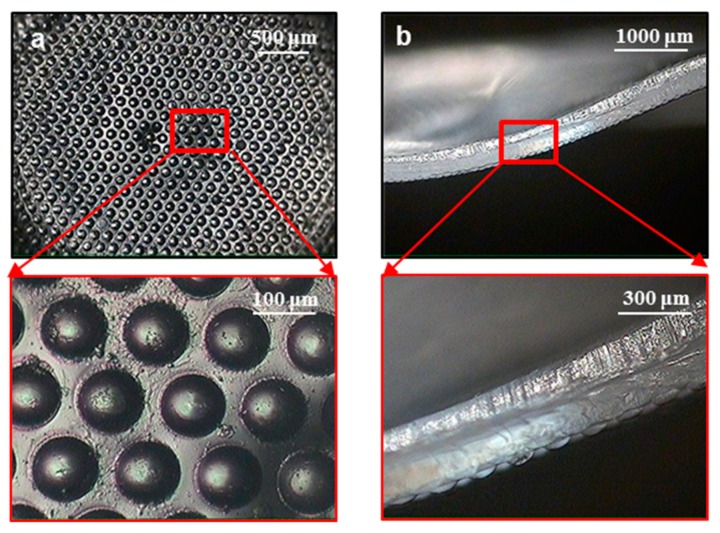
Microscopic images of the dot micropatterns on a curved surface: (**a**) bottom view; (**b**) sectional view.

Figure 9 shows microscopic images of the stretched film containing dot micropatterns. A Type 2 horn was also used in this ultrasonic stretching process. It can be seen that the micropatterns also retained their original shapes on the curved surface. The pattern heights after stretching were measured to be 26.3 ± 1.8 μm, which corresponds to 89.8% of the pattern height before stretching (29.3 μm). That is, the pattern height was reduced by 10.2% during the ultrasonic stretching process. This reduction in pattern height was caused by the contact pressure between the patterned film and the soft mold insert. However, the amount of reduction was as small as 10%–15%, and hence the reduced patterns are still expected to perform their functionality as micropatterns.

## 4. Discussion

**Figure 10:**
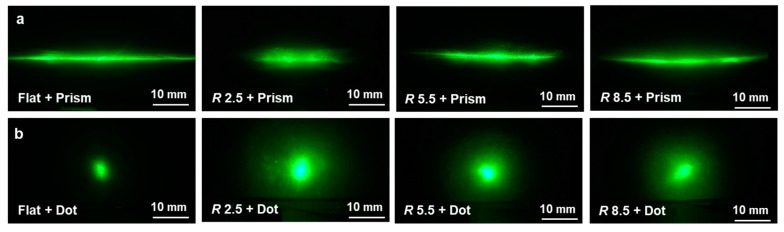
Comparison of light diffusion characteristics of curved polyethylene terephthalate (PET) films containing micropatterns: (**a**) prism pattern; (**b**) dot pattern.

To investigate the functionality of the curved films containing micropatterns, their optical diffusion and illumination properties were analyzed. Figure 10 compares images obtained using laser light, observed from behind patterned films with different curvatures and pattern types. Figure 10a shows the laser images through the prism-patterned films depending on the film curvature (i.e., the horn tip radius). It was observed that the flat patterned film caused the light diffusion along the horizontal direction because the prism patterns were arranged along the vertical direction. Although the other patterned films with different curvatures also showed horizontal diffusions, the amount of diffusion differed according to the film curvature—as the curvature increased (i.e., the horn radius decreased), the amount of horizontal diffusion was reduced while vertical diffusion was increased slightly. Figure 10b shows the laser images through the dot-patterned films according to the film curvature, which shows that the order of diffusion was increased as the curvature increased.

**Figure 11:**
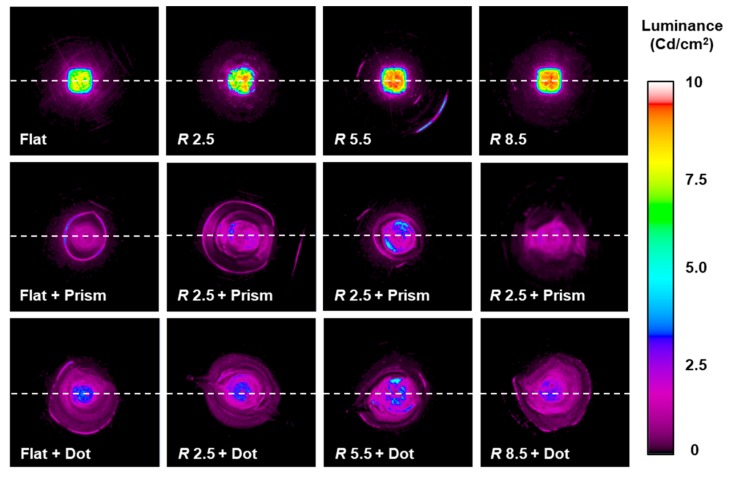
Comparison of light illumination characteristics of curved PET films with different curvatures and pattern types.

Figure 11 compares the distributions of luminance behind patterned films with different curvatures and pattern types. In this case, the luminance distributions of plain films with different curvatures were also measured. Overall, the luminance through the micropatterned films showed wider distribution, whereas their magnitudes decreased compared to the plain films. This can be explained by light scattering in the micropatterns as well as the degeneration of the transparency of the patterned region. On the other hand, increasing the film curvature increased the area of the illuminated region. These results indicate that the combined use of micropattern and curvature can effectively vary illumination characteristics.

## 5. Conclusions

In this study, an efficient micropatterning technology on a curved surface was developed by sequentially performing ultrasonic imprinting and ultrasonic stretching processes. Ultrasonic imprinting was performed to replicate micropatterns (prism and dot micropatterns) on a flat PET film. Ultrasonic stretching was then performed to create a curvature in the patterned film without damaging the micropatterns. For this purpose, a soft mold insert and three curved ultrasonic horns were used, and the relevant forming characteristics were analyzed experimentally. The experiments showed that the proposed two-step ultrasonic forming could develop micropatterns on a curved surface successfully. The change in the horn radius varied the curvature of the patterned film. This allowed us to adjust the functionality of the patterned film such as its light diffusion and illumination characteristics.

Compared to the previously developed patterning process for curved surfaces, the proposed two-step ultrasonic forming process has advantages: (i) a simple equipment setup that consists of a desktop-scale ultrasonic forming machine and a typical micropatterned stamp and (ii) a short cycle time of less than 20 s (10 s for ultrasonic imprinting and 7.3 s for ultrasonic stretching). Furthermore, the ultrasonic stretching process can also be used separately to form a patterned film that is fabricated by other micropatterning technologies. Further study is required to extend the proposed ultrasonic stretching process to form more complicated freeform surfaces.

## Figures and Tables

**Table 1:** Comparison-determined optimal dimensions for three horn types.

Horn Type	*R* (mm)	*D* (mm)	*r* (mm)
Type 1	2.5	24.8	6.8
Type 2	5.5	25.0	29.4
Type 3	8.5	25.8	71.3

**Table 2:** Comparison of vibration characteristics for the three ultrasonic horns.

Horn Type	*R* (mm)	*f* (Hz)	*δ_o_* (μm)	*γ*
Type 1	2.5	27,008	33.1 ± 1.0	2.02 ± 0.06
Type 2	5.5	26,989	33.4 ± 0.4	2.04 ± 0.02
Type 3	8.5	27,035	34.1 ± 0.8	2.08 ± 0.05

## Appendix A

Table A1, Table A2 and Table A3 provide CCD design tables for the three types of horns. For each horn type, nine FE analyses were performed with variations in the horn inlet diameter (*D*) and the corner radius (*r*). The output responses, natural frequency (*f*) and magnification ratio (*γ*), are also provided in Table A1, Table A2 and Table A3. These results were used in the response surface analyses to determine the optimal horn dimensions, as described in Section 2.4.

**Table A1:** CCD design table and the relevant FE analysis results (Type 1).

Design No.	*D* (mm)	*r* (mm)	*f* (Hz)	*γ*
1	25.0	6.5	26,973	2.15
2	24.0	6.0	26,958	1.98
3	26.0	6.0	26,891	2.32
4	24.0	7.0	27,025	1.98
5	26.0	7.0	26,994	2.32
6	25.0	5.8	26,910	2.15
7	26.4	6.5	26,930	2.39
8	25.0	7.2	27,029	2.15
9	23.6	6.5	26,998	1.91

**Table A2:** CCD design table and the relevant FE analysis results (Type 2).

Design No.	*D* (mm)	*r* (mm)	*f* (Hz)	*γ*
1	24.8	30.0	26,987	2.05
2	25.0	29.5	27,002	2.08
3	25.2	29.0	27,016	2.12
4	25.2	30.0	27,041	2.11
5	25.2	29.5	27,039	2.13
6	25.0	28.8	26,985	2.09
7	24.8	29.5	26,965	2.04
8	24.8	29.0	26,964	2.05
9	25.0	30.2	27,019	2.08

**Table A3:** CCD design table and the relevant FE analysis results (Type 3).

Design No.	*D* (mm)	*r* (mm)	*f* (Hz)	*γ*
1	25.0	71.5	26,822	2.00
2	25.0	70.8	26,813	2.00
3	24.0	71.0	26,599	1.87
4	26.4	71.5	27,144	2.18
5	25.0	72.2	26,830	1.99
6	26.0	71.0	27,041	2.13
7	26.0	72.0	27,055	2.12
8	24.0	72.0	26,609	1.86
9	23.6	71.5	26,518	1.81
